# Mental health first aid for eating disorders: pilot evaluation of a training program for the public

**DOI:** 10.1186/1471-244X-12-98

**Published:** 2012-08-02

**Authors:** Laura M Hart, Anthony F Jorm, Susan J Paxton

**Affiliations:** 1 Melbourne School of Population Health, University of Melbourne, Parkville, VIC, Australia; 2 School of Psychological Science, La Trobe University, Bundoora, VIC, Australia

## Abstract

**Background:**

Eating disorders cause significant burden that may be reduced by early and appropriate help-seeking. However, despite the availability of effective treatments, very few individuals with eating disorders seek treatment. Training in mental health first aid is known to be effective in increasing mental health literacy and supportive behaviours, in the social networks of individuals with mental health problems. Increases in these domains are thought to improve the likelihood that effective help is sought. However, the efficacy of mental health first aid for eating disorders has not been evaluated. The aim of this research was to examine whether specific training in mental health first aid for eating disorders was effective in changing knowledge, attitudes and behaviours towards people with eating disorders.

**Methods:**

A repeated measures, uncontrolled trial was conducted to establish proof of concept and provide guidance on the future design of a randomised controlled trial. Self-report questionnaires, administered at baseline, post-training and 6-month follow-up, assessed the effectiveness of the 4-hour, single session, mental health first aid training.

**Results:**

73 participants completed the training and all questionnaires. The training intervention was associated with statistically significant increases in problem recognition and knowledge of appropriate mental health first aid strategies, which were maintained at 6-month follow-up. Sustained significant changes in attitudes and behaviours were less clear. 20 participants reported providing assistance to someone with a suspected eating disorder, seven of whom sought professional help as a result of the first aid interaction. Results provided no evidence of a negative impact on participants or the individuals they provided assistance to.

**Conclusions:**

This research provides preliminary evidence for the use of training in mental health first aid as a suitable intervention for increasing community knowledge of and support for people with eating disorders to seek appropriate help.

**Trial registration:**

Australian New Zealand Clinical Trials Registry ACTRN12611001181998

## Background

Effective, evidence-based treatments are available for eating disorders. Current treatments for bulimia, binge eating disorder and their sub-threshold counterparts are associated with good long-term outcomes, as follow-up studies show a majority return to health and functioning [1-3]. While further research and development is needed for the treatment of anorexia [4-6], many individuals achieve lasting recovery, and improvements in social functioning and quality of life are possible with current interventions, even where recovery is not attained [7,8].

Despite the benefits of available treatments, only a minority of community members who experience an eating disorder seek appropriate care. It is estimated that less than one quarter of individuals with an eating disorder seek specific, evidence-based treatment [9]. Receiving treatment for weight-loss or another mental health problem, such as depression or anxiety, is much more common than receiving appropriate formal care for an eating pathology [10-13]. Those with eating disorders are also more likely to engage help-seeking from informal sources, such as their social network, or to use self-help strategies, such as increasing intake of vitamins and minerals or searching for information on the internet, than they are to obtain formal treatment [12,14-16]. While there are self-help strategies known to be useful in reducing some eating disorder symptoms, these are rarely utilised [9,17].

Given that disordered eating and exercising cause significant personal, social and economic cost, effective treatment interventions that reduce symptoms and their associated burden are imperative for individuals with eating disorders [18-20]. There is, therefore, an immediate and widespread need for interventions that decrease barriers and increase incentives for seeking appropriate care [13,21]. If the significant burden imposed by eating disorders is to be decreased, research focused on the development, implementation and evaluation of effective programs for increasing help-seeking, is desperately needed.

*Mental health literacy* has been defined as knowledge and beliefs about mental illness that aid their recognition, management or prevention [22]. Interventions that aim to improve mental health literacy in the social network of individuals with eating disorders provide a promising avenue for increased help-seeking, because of the important role family and friends can play in recognising an eating disorder, reducing stigmatising attitudes, overcoming illness related barriers and facilitating engagement with treatment [21,23,24]. The social network of individuals with eating disorders is known to greatly influence the decision to seek treatment [21] and provide support and motivation for recovery [25,26]. Increasing the capacity of the social network is therefore likely to be a more effective strategy than increasing the knowledge of individuals with eating disorders alone.

**Figure 1:**
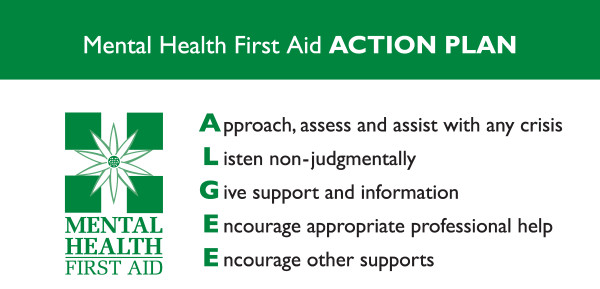
**ALGEE action plan for providing mental health first aid**. Mental health first aid techniques are taught in a 12-hour training program offered by the MHFA Training and Research Program. Like the successful D.R.A.B.C action plan for emergency medical first aid, the MHFA Training and Research program uses an action plan, ALGEE, to teach members of the public how to assist individuals with mental illness to manage symptoms and seek appropriate help.

One promising community-based intervention designed to increase mental health literacy is mental health first aid training. *Mental health first aid* is defined as the help provided to a person developing a mental health problem or experiencing a mental health crisis. The first aid is given until appropriate professional treatment is received, or the crisis resolves [27]. Mental health first aid techniques are taught in a 12-hour training program offered by Mental Health First Aid International (MHFA). MHFA was started in 2001 in response to Australian surveys revealing that the public lacked skills in responding to mental health problems [22,28,29]. Like the successful D.R.A.B.C action plan for emergency medical first aid, the MHFA training program utilises an action plan and provides information to the public about symptoms of various mental illnesses, their current effective treatments and how to assist individuals to manage symptoms and seek appropriate help (see Figure 1). MHFA has recently updated all of its training content to reflect the best practice consensus-based strategies developed over a series of Delphi studies [30-37]. MHFA courses are available in fifteen separate countries, including throughtout the United States, Canada, the United Kingdom, Europe and parts of Asia [38]. MHFA training courses have been well evaluated and found to be associated with changes in knowledge, attitude and behaviours [29,39-41]. Although a Delphi study has been completed to establish what the best practice strategies are for providing mental health first aid to someone with an eating disorder [42], prior to this research these recommendations had not yet been translated into a training intervention and their potential impact had not been evaluated. 

The aim of the current study was to examine whether mental health first aid training for eating disorders was effective in changing knowledge, attitudes and behaviours towards people with eating disorders. It was expected that the single session training intervention would be associated with an increase in participant mental health literacy, a decrease in participants’ stigmatising attitudes towards people with eating disorders, an increase in participants’ provision of supportive first aid behaviours towards those with eating disorders, and the promotion of help-seeking among those who were recipients of first aid.

## Methods

### Participants

A desired sample size of *n* = 84 was calculated based on a power analysis which assumed that, given no correlation between baseline and post-training scores, a sample of 64 participants would give 80% power to detect a medium effect size (*d* = 0.5) from baseline to post-training with alpha = 0.05 (*Sample Power 2.0*). This was increased to *n* = 84 to allow for 30% drop-out (*n* = 20) between baseline and follow-up.

Participants were recruited from the residential halls and colleges affiliated with The University of Melbourne; a large metropolitan campus with a diverse student population [43]. All undergraduate students residing on campus were eligible to participate. These young adults were chosen for convenience sampling because they represent a group at very high risk of developing an eating disorder [44,45], and a group who, given the nature of their residential environment, is likely to require skills in providing mental health first aid [46,47]. Welfare staff from campus residences were also included in the sample, as they were able to provide support to students who received the training, and were likely to be in contact with any individuals receiving treatment for an eating disorder.

Recruitment advertisements were posted as flyers on noticeboards around residences, presented orally at dinner meetings, or written in weekly news emails. The advertisements included information about eating disorders and MHFA training. Student and staff training sessions were run separately. Approval for this research was granted by the University of Melbourne Human Research Ethics Committee.

### Intervention

The training intervention was a 4-hour single-session, classroom-style, education program designed to be presented to groups of between 5 and 15 people. It included didactic teaching using a Powerpoint presentation, small-group learning activities (in pairs or threes) and whole-group discussion components. A teaching manual was developed to guide facilitation and ensure fidelity. Training was delivered by one primary instructor with a second facilitator assisting with group activities and participant questions. Both were trained and accredited MHFA Instructors [38].

The program content, presented across four consecutive units, is outlined in Table 1. The structure and content were developed based on the successful model devised by the MHFA program [29,39]. The information presented on symptoms, possible causes, effective evidence-based treatments and early intervention was gleaned from reviews of current scientific literature. The information on warning signs and first aid strategies was based on the previously developed guidelines document *Eating Disorders: First aid guidelines*[42,48]. The training protocol and materials were assessed by a working group of experts in eating disorders or MHFA training, and a pilot session was conducted, prior to commencement of the research. 

**Table 1:** Structure and content of the ‘Mental Health First Aid Training Course for Eating Disorders’ intervention

**Unit 1:**	**Unit 2:**	**Unit 3:**	**Unit 4:**
**Introduction**	**Eating Disorders**	**MHFA for eating disorders**	**Evaluation activity and conclusion**
Introductory activities	What are eating disorders?	First aid for crisis situations:	Post-training questionnaire
Common mental illnesses occurring in youth	Signs of a developing disorder	i. Medical emergencies	Question and answer time
Impact of mental illness	Importance of early intervention	ii. Suicidal thoughts and behaviours	Handing out of certificates and end matter
Youth mental health first aid	Risk factors for eating disorders	iii. Non-suicidal self-injury	
The MHFA action plan	MHFA for eating disorders – Action 1	MHFA for eating disorders – Actions 2-5	
1 hour	1 hour	1.5 hours	0.5 hours

### Measures

An assessment battery, including a range of self-report questionnaires, was designed to measure change in knowledge, attitudes and behaviours about eating disorders, as well as gather information about the demographic characteristics of participants and their mental health status.

Knowledge of eating disorder symptoms was assessed with a single item that asked participants to *give a brief description of what you think the main signs or symptoms of an eating disorder might be.* The open-ended responses were scored against the 25 ‘warning signs of a developing disorder’ outlined in *Eating Disorders: First aid guidelines*[48]; one point was awarded for each warning sign mentioned. The inter-rater reliability for scoring items was *r =* 0.94. This research was the first implementation of this measure.

Accurate problem recognition, knowledge of effective treatments and interventions, and attitudes towards bulimia, were assessed using the Mental Health Literacy Questionnaire for Bulimic Type Eating Disorders (MHLQ-B). The MHLQ-B was developed for the *Health and Well-Being* study by Mond and colleagues [49] and has been widely used in the study of eating disorder mental health literacy. The questionnaire begins with a vignette describing a fictional young adult woman ‘Kelly’ whose symptoms meet DSM-IV-TR diagnostic criteria for bulimia purging subtype.

A *knowledge of effective treatments and professionals scale* was constructed from the three questions on the MHLQ-B assessing knowledge of individuals who might be helpful (15 items), knowledge of treatments or activities (16 items) and knowledge of medications (eight items). Seven items were selected to construct the scale, based on the evidence in the current treatment literature, and the recommendations in the *Eating Disorders: First aid guidelines*[48], which suggest that psychologists, psychiatrists, dietitians/nutritionists, GPs, CBT, getting advice about diet and nutrition, and antidepressants, are all effective for eating disorders, and can therefore be considered ‘helpful’ for bulimia. Hence, participant responses were scored from zero (none of these items rated as ‘helpful’) to seven (all items rated as ‘helpful’). Although this approach has been used to assess knowledge of treatments for depression in previous evaluations of MHFA training [50], this research was the first time this measure had been used to assess knowledge of effective treatments and professionals for eating disorders.

A novel addition to the analyses of the MHLQ-B was the inclusion of the *knowledge of informal help-seeking scale*. Because there are informal activities known to facilitate formal treatment seeking or the management of disordered eating symptoms, the current research also sought to quantify how knowledge about broader help-seeking activities changes in response to the training program. From the 40-item list of individuals, treatments or activities, and medications, provided in the MHLQ-B, those that were recommended in the *Eating Disorders: First aid guidelines*[48], as appropriate and helpful, were used to construct the scale: friends, family members, using a self-help treatment manual and getting information about problem eating and available services. Hence, participant responses were scored from zero (none of these items rated as ‘helpful’) to four (all items rated as ‘helpful’).

The First Aid Knowledge Test (FAKT) was developed for use in the current research to test participant knowledge of best practice first aid strategies, as outlined in *Eating Disorders: First aid guidelines*[48]. It contains 26 true/false statements regarding knowledge and behaviours required for providing optimal mental health first aid. Responses are via a single forced-choice selection from the options: ‘Agree’, ‘Disagree’ or ‘Not sure’. One point is given for each answer that concurs with the information in the guidelines (i.e. an ‘agree’ response in relation to a true statement), resulting in a possible total score range of 0-26. The measure was validated with pilot testing.

The Social Distance Scale assesses levels of social rejection that members of the community are likely to impose on individuals with mental illness [51]. It comprises seven questions that ask how likely a participant is to react to a person with mental illness, as described by an accompanying vignette. Each question is rated on a 4-point Likert scale (1 = ‘definitely willing’ to 4 = ‘definitely unwilling’) [52]. A composite measure of social distance is calculated by adding across all items, with higher scores indicating a greater degree of desired social distance and more negative attitudes towards people with mental illness. In the current research possible total scores ranged from 0-35.

The Level of Contact Report [53] measures how familiar members of the public are (what level of contact they have) with individuals with mental illness. It was developed to examine whether level of familiarity had any effect on stigmatising attitudes, such as perceiving a person with schizophrenia as dangerous or unpredictable. The instrument lists 12 situations involving contact with an individual with mental illness. The highest score reported by a participant is taken as the total score. In the current research all items were modified to be specific to eating disorders, such that all references to ‘mental illness’ were replaced with ‘eating disorder’.

Throughout the questionnaire battery, six items were included to assess participants’ provision of mental health first aid to individuals with eating disorders, knowledge of the MHFA action plan and confidence in providing assistance. They were: (1) *In the last 6 months have you had contact with anyone who you think might have an eating disorder?* (2) *If ‘yes’, how many?* (3) *If you have had contact with someone who has an eating disorder, in the last 6 months, have you offered them any help?* (4) *If you offered help, what type of help was it?* (5) *If Kelly was someone you knew and cared about, how would you help her?* (6) *If you had contact with someone who had a problem like Kelly’s, how confident would you feel in helping them?* The items were based on those used in previous MHFA training evaluations [50,54-56]. Items 1, 3 and 6 required forced-choice responses, while items 2, 4 and 5 were open-ended. Responses to item 4 were coded into one of nine categories according to recurring themes. Category frequencies were then assessed across time points. Responses to item 5 were scored against the six components within the MHFA action plan (see Figure 1). Because the first action *A – approach the person, assess and assist with any crisis* contained many concepts, this was split into two categories (approach and assess/assist). The remaining four actions comprised one category each. For each of the six categories, responses were scored out of two points: a score of two was given if participants correctly described an action and provided specific detail about how they would undertake that action; a score of one was given if they mentioned the action but did not provide detail on how it may occur; and a score of zero was given if the action was not mentioned. A total score was calculated by adding together the scores for each of the categories, with a possible total score falling between 0-12. The inter-rater reliability on these items was *r =* 0.91. This scoring system has been used in previous research investigating MHFA action plan knowledge [40,57].

The First Aid Experiences Questionnaire was developed by Jorm, Kitchener and Mugford [56] for a follow-up study of participants who had attended MHFA training. It is designed to elicit open-ended information about helping behaviours towards individuals with mental health problems and about what the perceived effect of those behaviours was. The questionnaire has also been used in research investigating the utility and impact of accessing MHFA guidelines on the internet [58]. To assess whether the information provided in the training program was generalised to illnesses other than eating disorders, the questionnaire also asked whether participants had provided first aid to individuals who were experiencing mental health problems other than an eating disorder.

Given that some previous research has found that providing preventive interventions, which describe eating disorder symptoms, can lead to increases in eating pathology [59,60]*The Eating Disorders Examination Questionnaire* (EDE-Q) [61] was used in the current research to assess for any negative impact on participants’ eating pathology. The EDE-Q is currently considered the gold-standard for eating disorder research [62-66]. Australian research suggests that a global score of 2.8 reliably predicts clinical eating disorder status in females [65], however, a reliable cut-off has not yet been established for males.

The K10, a general measure of non-specific psychological distress, was also used to assess for any negative impact on participant psychopathology. Total scores range from 10-50, with higher scores indicating a higher level of distress [67].

### Design and analyses

The effectiveness of the training program was evaluated using an uncontrolled, repeated measures design. The assessment battery was administered at three time points: before the training course commenced (baseline), immediately after training concluded (post-training) and 6 months after the training program was completed (follow-up). Table 2 shows which measures were employed across each assessment time point. Because the measures of behaviour and mental health measured a time frame greater than one week, these were administered at baseline and follow-up only. The primary outcomes of interest were the problem recognition item within the MHLQ-B and the FAKT.

**Table 2:** Instruments administered to measure knowledge, attitudes behaviour and mental health status of participants, across time points

**Variable measured**	**Instrument**	**Baseline**	**Post-training**	**Follow-up**
Knowledge	Knowledge of Eating Disorder Symptoms	✓	✓	✓
	MHLQ-B			
	problem recognition	✓	✓	✓
	knowledge of effective treatments scale	✓	✓	✓
	knowledge of informal help-seeking scale	✓	✓	✓
	First Aid Knowledge Test	✓	✓	✓
	Mental Health First Aid Questions			
	item 5 - ALGEE	✓	✓	✓
Attitudes	Social Distance Scale	✓	✓	✓
	MHLQ-B			
	Beliefs about bulimia (3 items)	✓	✓	✓
Behaviour	Level of Contact Report	✓		✓
	Mental Health First Aid Questions			
	item 1 – Any contact	✓		✓
	item 2 – Number of contacts	✓		✓
	item 3 – Amount of help	✓		✓
	item 4 – Type of help	✓		✓
	item 6 – Confidence	✓	✓	✓
	First Aid Experiences Questionnaire			✓
Mental Health Status	EDE-Q	✓		✓
	K10	✓		✓

Repeated measures ANOVAs were used for continuous measures completed at all three time points. Where assumptions of sphericity where violated, the Hunyh-Feldt method for adjusting degrees of freedom was used [68]. Where the omnibus test was significant, planned contrasts were conducted to assess whether there was a significant change from baseline to post-training and baseline to follow-up. Dependent (paired) samples two-tailed *t*-tests were used for continuous measures completed at baseline and follow-up only. For ordinal data, Friedman’s ANOVA was used and Wilcoxon signed-rank tests were then conducted where the omnibus test was significant [68,69]. For dichotomous data, a Cochran’s Q test was used for *k* samples and the McNemar test for paired comparisons [68,69]. A Pearson’s chi-square analysis was conducted in the assessment of whether there were differences in first aid experiences between staff and students in the sample, because these groups were independent. Unless corrections for inflated error rate were required, all tests were conducted using α < .05. For open-ended responses, thematic analysis was used to section data into commonly occurring categories, which could then be assessed for frequency.

### Procedures

Prior to attending the training program, participants were sent an electronic link to the baseline questionnaire, hosted by an online survey software system (surveymonkey.com). Immediately after the training, participants completed the post-training questionnaire and an electronic link to the follow-up questionnaire was sent to each participant’s email address 182 days (6 months) after the date of attendance at the training program. Personalised email and SMS text prompts were sent at one week intervals, for a maximum of three weeks, to remind participants to complete the questionnaire.

## Results

### Participants

#### Characteristics at baseline

Ninety participants completed the baseline questionnaire. Participant characteristics are shown in Table 3. Participants ranged in age from 17 to 62 years (*M* = 23.84, *SD* = 10.43). Participants had studied at the tertiary level, on average, for 2.68 years (*SD* = 3.16). A very small number indicated they had received prior training in eating disorders or mental health, the majority of whom were staff acting in a welfare capacity at their college. Similarly, very few participants indicated that they had read the previously developed guidelines prior to attending the training.

**Table 3:** Characteristics of participants completing baseline and follow-up questionnaires

	**Baseline**		**Post-training**		**Follow-up**	
	**(n =90)**		**(*n* = 82)**		**(*n* = 73)**	
	**n**	**%**	**n**	**%**	**n**	**%**
Female	51	56.7	45	54.9	43	58.9
Staff	21	23.3	20	24.4	20	27.4
Student	69	76.7	62	75.6	53	72.6
Born in Australia	67	74.4	60	73.2	53	72.6
English second language	7	7.8	7	8.5	7	9.6
Attended MHFA course	5	5.6	5	6.1	5	6.9
Read ED guidelines	4	4.4	3	3.7	3	4.1
Attended ED course	5	5.6	5	6.1	5	6.9

The mean EDE-Q Global score for females in the sample was 1.52 (*SD* = 1.12) indicating normative eating pathology (e.g. *M* = 1.52, *SD* = 1.25) [70]. The score for males was 0.83 (*SD* = 0.70), indicating the sample scored slightly lower (less pathological) than normative (e.g. *M* = 1.09, *SD* = 1.00) [71]. This finding may be due to the inclusion of staff in the current sample, who were older than the sample used to establish norms, and the trend for EDE-Q scores to decrease with age [70]. Seven female participants scored above 2.80, suggesting they were likely to be experiencing a clinically significant eating disorder [63]. Cut-off scores for establishing probable diagnoses in community samples of males have not yet been reported. However, they are likely to be lower than that for females. No males scored above 2.8, although, two scored above 2.50.

The mean total K10 score was 18.1 (*SD* = 4.78), indicating scores were higher than normative (e.g. M = 14.20) [67]. Nine participants scored above 25; according to Australian national survey data, 38% of individuals who score in this range will experience a clinically significant affective disorder [67].

#### Participant flow

**Figure 2:**
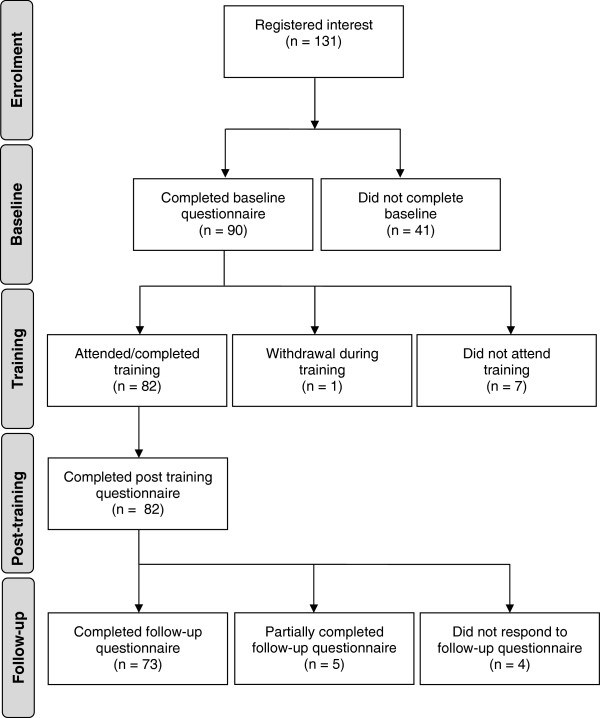
**Participant flow through research stages.** 90 participants completed the baseline questionnaire. 91% of these participants went on to complete both the training and the post-training questionnaire. No statistically significant differences were found at baseline, between those participants who completed the training and the post-training questionnaire, and those who did not. 73 participants (81% of baseline) completed the follow-up questionnaire 6-months after training. Five participants began the follow-up questionnaire but completed less than 50%. There were no participants who responded to more than 50% but less than 100% of the questionnaire. No statistically significant differences were found at baseline between those participants who completed the follow-up questionnaire and those who did not. Participants who did not complete the post-training or follow-up questionnaires were excluded from further analyses.

Flow of participants through the research stages is shown in Figure 2. Ninety-one percent of the 90 participants who completed the baseline questionnaire went on to complete the training and the post-training questionnaire. No statistically significant differences were found at baseline between those participants who completed the training and the post-training questionnaire and those who did not. Eighty-one percent of baseline participants went on to complete all three questionnaires. There were also no statistically significant differences found at baseline between those participants who completed the follow-up questionnaire and those who did not. Because of the small number who failed to complete all three stages of the evaluation (*n* = 17), and the lack of any significant difference between completers and drop-outs, the participants who did not complete the post-training or follow-up questionnaires were excluded from further analyses.

### Knowledge

Results for the instruments assessing participant knowledge are shown in Table 4. Mean scores on knowledge of eating disorder symptoms remained stable over time and no significant difference was found between scores at baseline, post-training and follow-up.

**Table 4:** Results for instruments assessing changes in knowledge and attitudes

**Instrument**	**Baseline**	**Post-training**	**Follow-up**	**Sig Test**	**Change Baseline to Post-training**	**Change Baseline to Follow-up**
**Knowledge of Eating Disorder Symptoms** (mean total score)	3.4	3.4	3.1	*p* = .4		
**MHLQ-B**						
Problem recognised as BN (%)	17.8	42.5	28.8	*p* = .001	*p* < .001	*p* = .115
Problem recognised as any ED^A^ (%)	45.2	78.1	64.4	*p* < .001	*p* < .001	*p* = .007
Problem recognised as general mental health problem^B^ (%)	49.3	21.9	27.4	*p* < .001	*p* < .001	*p =* .002
knowledge of effective treatments scale^C^ (mean total score)	4.7	6.4	5.4	*p* < .001	*p* < .001	*p* < .001
knowledge of informal help-seeking scale (mean total score)	2.8	3.2	3.0	*p =* .007	*p =* .004	*p =* .054
**First Aid Knowledge Test** (mean total score)	16.1	23.2	20.4	*p* < .001	*p <* .001	*p <* .001
**Mental Health First Aid Questions**						
item 5 - ALGEE (mean total score)	2.0	4.0	3.0	*p* < .001	*p <* .001	*p =* .089
**Social Distance Scale** (mean total score)	16.0	16.2	15.6			
**MHLQ-B**						
*How distressing do you think it would be to have Kelly's problem?*	*p =* .51					
Extremely (%)	38.4	39.7	43.8			
Very (%)	53.4	56.2	49.3			
Moderately (%)	8.2	4.1	6.8			
A little (%)	0.0	0.0	0.0			
Not at all (%)	0.0	0.0	0.0			
*How sympathetic would you be towards someone with Kelly's problem?*	*p =* .002	*p* = .001	*p* = .17			
Extremely (%)	24.7	39.7	30.1			
Very (%)	56.2	50.7	53.4			
Moderately (%)	16.4	9.6	16.4			
A little (%)	2.7	0.0	0.0			
Not at all (%)	0.0	0.0	0.0			
*Have you ever thought it might not be too bad to be like Kelly?*	*p =* .07					
Always (%)	1.4	0.0	1.4			
Often (%)	6.8	5.5	6.8			
Occasionally (%)	19.2	16.4	19.2			
Rarely (%)	30.1	30.1	31.5			
Never (%)	42.5	47.9	41.1			

^A^ Any ED included use of the labels 'bulimia nervosa', 'anorexia nervosa' or 'a binge eating disorder or problem'.

^B^ Any mental health problem included use of the labels 'an anxiety disorder or problem', 'mental illness', 'depression' or 'low self-esteem or lack of self-worth'.

^C^ This included items that were considered to have evidence for efficacy or recommended in treatment guidelines for BN: antidepressants, cognitive behavioural therapy, getting advice about diet or nutrition, GP, psychiatrist, psychologist.

When asked what they thought was ‘Kelly’s main problem’ the majority of participants recognised the symptoms in the vignette as relating to a general mental health problem such as ‘low self-esteem or lack of confidence’ or ‘mental illness’. Although many correctly recognised Kelly’s problem at baseline as ‘bulimia nervosa’, the proportion increased significantly from baseline to post-training. However, this dropped at follow-up and was no longer significantly different from baseline. To assess whether recognition of the problem as any eating disorder changed over time, the proportion of participants responding ‘bulimia nervosa’, ‘a binge eating disorder or problem’ or ‘anorexia nervosa’, were assessed together. The proportion recognising the problem as any eating disorder increased significantly from baseline to post-training, and despite a fall at follow-up, was still significantly different to baseline levels. To assess whether recognition of the problem in the vignette, as a general mental health problem, changed over time, the frequency of all other responses representing a mental health related condition were assessed together (‘an anxiety disorder or problem’, ‘mental illness’, ‘depression’, ‘low self-esteem or lack of self-confidence’). There was a significant decrease in frequency from baseline to post-training and this was maintained at follow-up.

At baseline, participants scored an average of 4.7 points out of 7 on the *Knowledge of effective treatments and professionals scale*. Scores were significantly different across time points, with both post-training and follow-up scores being significantly higher than baseline. On the *Knowledge of informal help-seeking scale* participants scored an average of 2.8 out of 4. Again, scores were significantly different across time points, with post-training scores being significantly higher than baseline, however, after a decline in scores at follow-up these were no longer significantly different from baseline.

At baseline participants scored an average of 16.1 of a possible 26 on the FAKT. Scores were significantly different across time points, with both post-training and follow-up scores a significant improvement on baseline.

The fifth Mental Health First Aid item asked participants how they would help someone like Kelly. Average ALGEE action plan scores were significantly different across time points, however, while post-training scores were a significant improvement on baseline, this was not maintained at follow-up and scores were no longer significantly different from baseline.

### Attitudes

Results for the instruments assessing participant attitudes are also shown in Table 4. Scores on the Social Distance Scale remained stable and were not significantly different across time points. Participant attitudes towards bulimia were assessed via three items on the MHLQ-B. Responses to the first item *How distressing do you think it would be to have Kelly’s problem?* showed that a large majority of participants (>90%), across all three time points, believed it would be either ‘very’ or ‘extremely’ distressing to have Kelly’s problem. Although the percentage of participants who believed it would be extremely distressing, increased from 38% at baseline to 40% at post-training, then to 44% at follow-up, the ombnibus significance test found no significant differences in scores over time.

Responses to the item *How sympathetic would you be towards someone with Kelly’s problem?* revealed that the majority of participants, across all three time points, believed they would be ‘very’ or ‘extremely’ sympathetic towards someone with Kelly’s problem. Significant differences were found in responses over time. However, while there was a significant change in ratings from baseline to post-training, a comparison of baseline and follow up revealed no significant change. That the ratings of ‘extremely’ increased and ratings of ‘moderately’ decreased from baseline to post-training, though returned to baseline levels at follow-up, may have largely accounted for this finding.

Responses to the item *Have you ever thought it might not be too bad to be like Kelly, given that she has been able to lose a lot of weight?* showed that majority of participants reported that they had ‘never’ or ‘rarely’ thought it desirable to be like Kelly. These responses appeared stable over time and an omnibus test revealed no significant differences in ratings across the time points.

### Behaviours

Results for the instruments assessing participant behaviors are shown in Table 5. At baseline, the mean Level of Contact Report score was 7.49 of a possible 12, indicating that participants had experienced a medium level of contact with individuals with eating disorders. Significance testing revealed that there were no significant changes in scores over time.

**Table 5:** Results for instruments assessing changes in behaviour

**Instrument**	**Baseline**	**Follow-up**	**Sig Test**
**Level of Contact Report** (mean highest score)	7.49	7.57	*p* = .783
**Mental Health First Aid Questions**			
* item 1 – Any contact*			*p* = .768
Yes (%)	60	56	
No (%)	26	30	
Not sure (%)	14	14	
* item 2 – Number of contacts*^*A*^			*p* = .95
Contact with 1 person (n,%)	19, 47.5	13, 31.7	
Contact with 2 people (n,%)	11, 27.5	16, 39.0	
Contact with 3 people (n,%)	7, 17.5	10, 24.4	
Contact with 4 or more people (n,%)	3, 7.5	2, 4.9	
* item 3 – Amount of help*^*A*^			*p* = .08
No help provided (n,%)	17, 38.6	11, 26.8	
A little (n,%)	12, 27.3	15, 36.6	
Some (n,%)	12, 27.3	12, 29.3	
A lot (n,%)	3, 6.8	3, 7.3	
* item 4 – Type of help*^*B*^			
Asked someone more appropriate than myself to help the person (n,%)	3, 10.7	8, 25.8	*p* = .18
Talked to the person directly (n,%)	15, 53.	15, 48.4	*p* = 1.0
Offered general support (n,%)	6, 21.4	3, 9.7	*p* = .45
Offered information about illness/services (n,%)	3, 10.7	5, 16.1	*p* = .72
Encouraged/assisted with seeking prof help (n,%)	4, 14.3	9, 29.0	*p* = .23
Encouraged self help (n,%)	1, 3.6	4, 12.9	*p* = .38
Offered practical help (n,%)	6, 21.4	2, 6.5	*p* = .29
Offered emotional support (n,%)	11, 39.3	8, 25.8	*p* = .61
Risk assessment/monitoring (n,%)	1, 3.6	4, 12.9	*p* = .38
* item 6 – Confidence in helping Kelly (mean score)*	2.2	2.7	*p* < .001
Not at all (n,%)	16, 21.9	3, 4.1	
A little (n,%)	34, 46.6	27, 37	
Moderately (n,%)	17, 23.3	34, 46.6	
Quite a bit (n,%)	6, 8.2	9, 12.3	
Extremely (n,%)	0, 0.0	0, 0.0	

^A^ = Data is for 44 participants at baseline and 41 at follow-up (ie. Only those who reported having had contact with someone with an ED).

^B^ = Data is for 28 participants at baseline and 31 at follow-up (ie. Not all of those who reported contact also offered assistance. This data is only for those who offered assistance).

In response to the question *In the last 6 months have you had contact with anyone who you think might have an eating disorder?* 60% said ‘yes’ at baseline. The modal number of contacts was 1 person (*n* = 19), and the total number of contacts reported by participants was 78. At follow-up, despite a small drop in the frequency of participants reporting that they had contact with someone with an eating disorder (56%), the modal number was 2 people (*n* = 16), and the total number of contacts was 97. Twenty-two participants reported an increase in the amount of contact across time. These changes, however, were not statistically significant. In addition, no statistically significant changes were found between baseline and follow-up for the items assessing the amount of assistance given and the type of assistance given.

Results for the final Mental Health First Aid question regarding participant confidence revealed that confidence ratings were significantly different across time points, with both post-training, and follow-up ratings being significantly higher than baseline.

To assess the qualitative nature of first aid interactions participants had with individuals with eating disorders, the *First Aid Experiences Questionnaire* asked a number of open-ended questions about their intervention. Of the 73 participants in the total sample, 41% (*n* = 30) of participants provided feedback on their first aid experiences. Those who did not report on a first aid experience (*n* = 43, 59%) were asked a series of open-ended questions about what they would do in the future if a situation arose. Responses to these are shown in Table 6. For those who did experience a first aid situation a different series of questions, about whether the participant had been able to assist the person and how they felt the intervention had gone, were presented. Responses to these questions are shown in Table 7. A full description of the qualitative data, including descriptions of first aid provided to individuals experiencing a mental health problem other than an eating disorder, is given in Additional file 1: Document 1.

**Table 6:** Responses to the First Aid Experiences Questionnaire for participants who did not report experiencing a first aid situation (n = 43)

	**n**	**%**
Q1. Is this what you would have expected, or is it somewhat surprising not to have come across such a situation?*		
Surprised	17	39.5
Had expected to encounter someone with an eating disorder	17	39.5
Had been in contact, but didn’t provide first aid because we were not good enough friends	8	18.6
Had been in contact with someone with an ED before the training and this continued after the training	3	7.0
Q2. In the future, if you were to come across someone who you believed was experiencing an eating disorder, how well prepared would you feel to deal with the situation?*		
Very well, or well prepared	33	76.7
Prepared	9	20.9
Prepared but uneasy	5	11.6
Not at all prepared	1	2.3
Q3. How has attending 'Mental Health First Aid Training Course for Eating Disorders' changed how you relate to or feel about people who experience eating disorders?*		
Know more about EDs	15	34.9
Know more/more confident about how to help	14	32.6
More empathy for those with EDs	14	32.6
No change because of prior expertise	2	4.7
No change	4	9.3

* Responses were open-ended and therefore participants may have mentioned multiple ideas/categories.

**Table 7:** Responses to the First Aid Experiences Questionnaire for participants who did report experiencing a first aid situation (n = 30)

	**n**	**%**
*Q1. In what capacity do you attend the college?*		
Student	22	73.3
Staff	7	26.7
Q2. Could you tell us something about the situation(s) and the problem(s) you believed the person was experiencing?*		
Change in eating habits (ate less/restricted diet)	15	50.0
Binge eating	2	6.7
Change in exercise patterns	11	36.7
Vomiting	1	3.3
Weight loss	17	56.7
*Q3. Did you try to assist the person you thought might be developing or experiencing an eating disorder?*		
No	10	33.3
Yes	20	66.7
Q4. What was the reason(s) that you were not able to assist that person?* (n = 10)		
Not close enough to the person/others more appropriate to provide first aid	5	50.0
Person already adequate receiving support/care	4	40.0
Assistance refused	1	10.0
Q5. Can you give us an example of something you did to assist the person?* (n = 20)		
Approached the person, discussed my concerns, listened to them	11	55.0
Discussed my concerns with someone more appropriate to provide first aid	7	35.0
Offered information and resources about EDs	2	10.0
Encouraged the person to seek help	6	30.0
Encouraged the person to use other supports	5	25.0
*Q6. When assisting the person did you use the information provided in the 'Mental Health First Aid Training Course for Eating Disorders'? (n = 20)*		
Yes	14	70.0
No	0	0.0
Not sure	6	30.0
*Q7. How successful do you think you were in assisting the person?(n = 20)*		
Very successful	0	0.0
Successful	9	45.0
Neither successful nor unsuccessful	11	55.0
Unsuccessful	0	0.0
Very unsuccessful	0	0.0
Q8. Would you like to comment on what happened?* (n = 20)		
Unaware of outcome because I didn’t provide the first aid	2	10.0
The person received professional help	3	15.0
The person showed some signs of recovery	3	15.0
The person had made some changes, but am unsure how much recovery is progressing	3	15.0
*Q9. Do you think the information in the 'Mental Health First Aid Training Course for Eating Disorders' contributed to the level of success you had in assisting the person? (n = 20)*		
Very much	6	30.0
A little bit	12	60.0
Not sure	1	5.0
Not really	1	5.0
Definitely not	0	0.0
*Q10. When assisting the person, did you do anything differently from what you would have done before attending the 'Mental Health First Aid Training Course for Eating Disorders'? (n = 20)*		
Yes	11	55.0
No	4	20.0
Not sure	5	25.0
Q11. If you answered 'yes' please provide a short description* (n = 11)		
Changed the way I approached the person	7	63.6
Because I had more knowledge I noticed more symptoms	4	36.4
Suggested professional help though wouldn’t have before	3	27.3
More understanding and supportive of the person	2	18.2
*Q12. Did you suggest to the person you were assisting that they should seek professional mental health care (*e.g. *from a GP, psychologist, psychiatrist) (n = 20)*		
Yes	10	50.0
No	3	15.0
The person was already receiving mental health care	3	15.0
Not sure	4	20.0
*Q13. As a result, did the person you were assisting seek mental health care from a professional? (n = 20)*		
Not applicable	6	30.0
Yes	7	35.0
No	0	0.0
Not sure	7	35.0

* Responses were open-ended and therefore participants may have mentioned multiple ideas/categories.

### Mental health status

A statistically significant reduction in the mean EDE-Q global score was found between baseline (*M* = 1.2) and follow-up (*M* = 1.1), indicating a reduction in eating disorder psychopathology *t*(72) = 2.27, *p* = .026.

The mean total K10 score dropped slightly from baseline (*M* = 18.1) to follow-up (*M* = 17.2), though no statistically significant differences were found across the two time points *t*(72) = 1.53, *p* = .129.

## Discussion

The aim of the current study was to examine whether a training intervention on mental health first aid for eating disorders was effective in changing knowledge, attitudes and behaviours towards people with eating disorders. Results suggest that the training intervention was associated with significant improvements in knowledge, which were maintained over time. There was no evidence to suggest the training program produced sustained changes in attitudes towards individuals with eating disorders, though these were relatively empathic at baseline. There was also little quantitative evidence to suggest the training program produced significant changes in behaviours towards individuals with eating disorders, however, qualitative responses in the small sample who did have contact with an individual with an eating disorder, suggested the training was associated with changes in first aid behaviours.

### Changes in knowledge

There was no significant change in the knowledge of eating disorder symptoms across time. It is possible that this finding is the result of participants lodging less detailed or less specific answers across time, as the follow-up questionnaire was sent to the majority of participants between October and November; a busy exam period and the end of the academic year. It is also possible that the training failed to produce a significant increase in knowledge scores over time because there was not enough emphasis placed on eating disorder specific signs and symptoms throughout the curriculum. In fact, the training emphasises the importance of recognising the development of non-specific signs of distress, as this is the best opportunity to provide timely mental health first aid. As such, participants were taught to look for a change in “a person’s thinking, emotional state and behaviour, which disrupts the ability to work or carry out other daily activities and engage in satisfying personal relationships” [27], which is how the MHFA program defines the onset of a mental health problem. Future evaluations may therefore benefit from an item designed to measure a change in knowledge regarding the most appropriate time to provide mental health first aid, rather than specific knowledge relating to the wide range of eating disorder signs and symptoms.

Unlike knowledge of signs and symptoms, accurate recognition of the problem in the MHLQ-B vignette, as ‘bulimia nervosa’, increased significantly immediately after the training. Furthermore, recognition of the problem as any eating disorder also increased, and this change was sustained over time. Importantly, this effect of increased recognition, was not generalised to any mental health problem; participants were not more likely to label the problem in the vignette with general terms relating to mental ill-health at follow-up, indicating that the improved recognition was specific to eating disorders, and in particular to bulimia. It is interesting that participants’ increased recognition of the problem as any eating disorder was more robust than the specific recognition of the problem as bulimia. Given that the training encourages participants to look for *changes* in eating and exercise behaviours that are interfering with function, rather than encouraging participants to look for indicators of diagnostic categories, this result is perhaps more desirable than having a sustained increase in the specific recognition of the problem as bulimia.

The *Knowledge of effective treatments and professionals* scale in the MHLQ-B showed that participants’ knowledge of effective treatments for eating disorders also significantly improved over time. Unfortunately this was not true for the *Knowledge of informal help-seeking scale*. While it was found that total scale scores significantly improved after the training, these improvements were not maintained at follow-up. This result, however, is perhaps not unexpected. While the treatment literature recognises the importance of friends and family in the help-seeking and recovery process, it is also apparent that the social network can have a negative influence on the development of mental illness and barriers to care [72,73]. It is therefore true that the effectiveness of individuals in facilitating help-seeking and recovery will depend on their level of knowledge, skill and empathy. As this concept was discussed as part of the training, it is perhaps not a surprise that ‘helpful’ ratings did not change across time. It is also important to acknowledge that this study is the first time the *Knowledge of informal help-seeking scale* had been implemented. Given its construction from just 4-items, further testing of its psychometric properties would further elucidate its utility in future evaluations of MHFA training. Furthermore, future evaluations may benefit from an examination of changes to inappropriate informal help-seeking, rather than a narrow focus on changes in the small number of appropriate strategies.

The FAKT showed that participant knowledge of first aid skills improved significantly after the training and, despite a drop-off in the follow-up period, this improvement was maintained at follow-up, indicating that the training was associated with a gain in knowledge that was sustained over time. As this is the first time the FAKT instrument has been implemented, the psychometric properties are not well known. It is therefore possible that increases from baseline to post-training were the result of re-test effects. However, given that there was a small drop in knowledge from post-training to follow-up, it appears that each re-testing event does not necessarily result in ongoing improvement over time.

Responses to the Mental Health First Aid item, which asked participants how they would help ‘someone like Kelly’, indicated that the training was associated with a significant increase in knowledge of the appropriate actions a person should take when providing a first aid intervention, as outlined by the MHFA action plan. However, this change was not sustained over time. There are two possible explanations for this result. One is that, given the open-ended response format for this item, participants provided less detailed responses at follow-up, due to their academic time constraints, and hence scored more poorly than at post-training. Another plausible explanation is that the training was insufficient in duration to produce a lasting effect. It is perhaps not unsurprising that participants could not remember these five specific actions, six months after receiving a 4-hour intervention. Furthermore, given that a previous evaluation of the full MHFA training program found an increase in knowledge of the action plan, which was maintained at six month follow-up [57], it is likely that had the current training involved more time to discuss and role-play the action plan, retention of information would have been maintained over time.

Overall, results for the items assessing knowledge showed that the training was associated with an immediate and lasting improvement in eating disorder problem recognition, knowledge of effective treatments, and consensus-based first aid strategies. Although results showed that there were increases in knowledge for informal help-seeking strategies and the MHFA action plan, these changes were not maintained over time, perhaps indicating that the intervention was too brief to produce lasting effects. These results do however provide preliminary support for the impact MHFA training can have on mental health literacy for eating disorders.

### Changes in attitudes

Scores on the Social Distance Scale remained unchanged after the training and during the follow-up period, compared to baseline. Interestingly, in a review performed by Jorm and Oh [74], interventions aimed at reducing stigmatising attitudes of similar length to the current training, were found to have similar results whereby no significant changes in the total score were found over time. It therefore appears that the current intervention may have been too short and required more direct contact with people affected by eating disorders, to achieve the desired reduction in social distance.

The MHLQ-B item assessing participant ratings of how distressing they believed Kelly’s problem would be, found that there were no significant differences associated with the training, despite some small fluctuations in ratings. Two plausible explanations for this result can been considered. First, it is possible that there was an insufficient ‘dosing’ effect, whereby the intervention was too short to provide lasting effects over time. If this were true, a longer intervention or booster session should produce the desired result. The second possibility is that participants were at ceiling when first measured at baseline. Given the high proportion of participants who rated Kelly’s problem as either ‘very’ or ‘extremely’ distressing, it is possible that the intervention was unable to produce any further increase. As a point of comparison, a community-based survey of adolescent girls’ mental health literacy for eating disorders found that most respondents believed that it would be ‘very’ (40.0%) or ‘extremely’ distressing (45.0%) to have a problem like Kelly’s, indicating that there was little need for an intervention to educate participants about the distressing nature of the condition [75].

The desirability of bulimic symptoms was also assessed with a MHLQ-B item, which found that the majority of participants reported that they had ‘never’ or ‘rarely’ thought that ‘it might not be too bad to be like Kelly given that she has been able to lose a lot of weight’. This finding is in contrast to previous evaluations of MHLQ-B, which have found much higher levels of desirability. The current finding may be an artefact of older adults and male participants being included in the sample, rather than a non-representative sample per se. That the desirability ratings remained stable over time and showed no significant change after the training, can be explained in two ways. First it is possible that the training had no effect on how desirable participants found bulimic symptoms. However, it is also possible that there was a ceiling effect. Given that it is expected the intervention would have the strongest effect among participants who considered bulimia desirable, though most did not indicate any desirability for bulimic symptoms at baseline, the beliefs of the current sample left little room for improvement.

Taken together, items assessing attitudes towards bulimia indicate that the sample at baseline were already empathic. It is therefore difficult to infer from these results whether the intervention was effective in changing attitudes about eating disorders. Although a sample with less empathic attitudes at baseline may have shown a statistically significant improvement after training, it remains a welcome finding that members of the community already express empathic attitudes towards individuals with eating disorders.

### Changes in behaviour

The amount of contact participants had with individuals with eating disorders was measured using the Level of Contact Report and two Mental Health First Aid questions. Across all measures, there were no significant differences found between the amount of contact with individuals with eating disorders before the training and the amount of contact after the training. One possible explanation for this finding is that the environment in which the participants studied, worked and resided, was not amenable to producing a change in the amount of contact a participant may experience. For example, the large majority of students and staff undertake residence at a college for an academic year, beginning at the start of March and ending in late November. Between these months, there is very little change in the composition of staff or students in the residences. In the current study, the baseline measurement, training and follow-up period all fell within the 2010 academic year. Furthermore, because college residences are an environment where individuals live in very close contact with one another, and the welfare of students is closely monitored by pastoral care teams, it is possible that the majority of individuals with eating disorders, who were present within the residences, were already identified at baseline and this number could not be significantly improved upon after the training and during the follow-up period. Another possible explanation for the non-significant change in contact is that the training intervention had no effect on participant behaviour toward those with eating disorders. However, given that there was a significant increase in the ability to accurately recognise an eating disorder, that knowledge of effective first aid strategies significantly increased, and there was no increase in social distance, it appears unlikely that the intervention would not influence contact to some degree. A second and longer follow-up period, crossing over more than one academic year, may elucidate any effect of the college environment on the level of contact found among participants. However, it should be noted that some previous evaluations of MHFA training have also recorded no change in the level of contact with those with mental illness [50,54].

The third Mental Health First Aid item was designed to measure change in the amount of help provided to those with eating disorders. No significant increase was found after the training. Some previous MHFA training evaluations have also failed to find an increase in amount of help provided. Of the four studies that have used the same method of evaluating help provided, two studies found no significant differences between baseline and follow-up [56,76].

The current study also found no change in the type of help provided by participants. Given the relatively small number of participants who reported providing first aid, it is possible that there was inadequate power to detect an effect. This postulate is supported by the finding that the majority of participants who provided first aid reported that they had done things differently to before they received the training, according to responses on the First Aid Experiences Questionnaire. Furthermore, the training was associated with a significant increase in participant confidence in providing first aid. In combination then, these results suggest that if a second and longer follow-up testing period was employed, which would allow participants more time to be in contact with and provide assistance to individuals with eating disorders, the existence of any significant changes in behaviour after the training, would be clarified.

Responses to the First Aid Experiences Questionnaire were largely positive, as many indicated that the training allowed them to feel more knowledgeable, confident and better prepared to recognise and provide assistance to someone developing or experiencing an eating disorder. Although the quantitative measures found no statistically significant increases in the amount of help provided, the open-ended information gathered by this instrument suggests that participants approached someone they were concerned about with more empathy and patience than before, and that this change was a result of the training. Furthermore, the majority of those who had not provided first aid indicated that the training had impacted on the way they viewed eating disorders as mental illnesses worthy of care, understanding and effective treatment. Interestingly, it appeared that the training generalised to assisting individuals with other mental health problems, as an equal proportion of participants reported providing first aid to individuals with mental illnesses other than eating disorders.

Participants who had provided first aid reported feeling reasonably successful in their intervention, however, many went on to explain that the unresolved nature of the person’s illness contributed to their sense that their intervention was not a complete success. Given that this finding reiterates that of the earlier evaluation of guidelines being downloaded from the internet [58], future MHFA training would do well to include a discussion of what ‘successful’ first aid might look like and how participants should not expect immediate or necessarily complete recovery, as a result of providing assistance and facilitating appropriate treatment seeking. Interestingly, a much larger proportion of participants who reported assisting someone with another mental health problem reported feeling that their first aid intervention was successful. The ambivalence about success may therefore be particular to assisting in the case of an eating disorder.

Importantly, the First Aid Experiences Questionnaire provided no reports of adverse experiences associated with an attempted first aid intervention. Conversely, participants indicated that as a result of their suggestion and assistance, seven individuals had sought help for their suspected eating disorder, and an additional three were better supported whilst already receiving care. Furthermore, three participants mentioned assessing for suicide risk where they wouldn’t have done so before the training. While statistical inferences from these data are not possible, it is encouraging that these responses indicate the training was associated with a higher level of effective assistance, and ultimately, more appropriate help-seeking.

In sum, the findings from instruments measuring behavior change suggest that there is some limited evidence for a change in first aid behaviours and an increase in appropriate help-seeking, albeit among a small number of participants. Many of the findings in the current research confer with those of previous MHFA evaluations employing a six month follow-up period, which suggests that a second and longer follow-up period, for example at 12 months after training, might allow for an increase in the size of the sample providing assistance, and thereby an increase in the power to detect any statistically significant effect of the training on behaviour change.

### Changes in mental health

The EDE-Q and K10 were implemented to assess any changes in the mental health of participants. Given previous research evaluating preventive interventions for eating disorders has found that providing information about eating disorder symptoms can lead to an increase in eating pathology, the EDE-Q was used in the current study to assess for any negative impact on participants. Despite being normative at baseline, the sample’s average global EDE-Q score was found to be significantly lower at follow-up. There are two possible explanations for this outcome. The first is that the intervention had a positive effect on eating pathology. The second is that the result is due to a re-test effect, whereby scores improved (decreased) with each testing occasion, as has been shown to be possible with psychiatric instruments designed to assess negative self-characteristics [77]. Without the presence of a control group to assess whether there is an inherent decrease in scores on the EDE-Q across time, it is not possible to conclude which of the two explanations is the more plausible. In any case, the current findings suggest no evidence that the training had a negative impact on the eating pathology of participants.

Although there was a slight decrease in K10 total scores from baseline to follow-up, this was not statistically significant. This finding is in accordance with previous evaluations of MHFA training and other mental health literacy interventions, and suggests that the training does not have a negative impact on the psychological distress of participants.

### Limitations

The absence of a control group is the primary limitation of this research. Without being able to control for extraneous variability in scores over time, concrete conclusions about the role of the training in causing improvements on a number of measures, such as the FAKT and EDE-Q, cannot be reached. However, given that the current research was designed to be a preliminary exploration of how the concept of mental health first aid might be applied to eating disorders, this study has produced some initial evidence to suggest that providing a training intervention to community members increases knowledge and confidence in providing first aid, as well as important data about how future evaluations could be best implemented.

A second limitation of this research is the composition of the sample at baseline. Prior to training, the majority of participants upheld empathic attitudes towards people with bulimia, had relatively good mental health literacy, a large proportion were already in contact with an individual with an eating disorder and had provided some level of assistance. In contrast to the community studies completed by Mond and colleagues, it appears as though the current sample were already functioning above average on a number of variables [e.g. [46,49,75,78]. It was perhaps the nature of the residential college environment, where students live in close contact with one another, members of staff are highly trained in providing pastoral care, and all are well educated, which resulted in participants scoring relatively highly on most measures at baseline. It is also possible that because recruitment materials contained information about both eating disorders and mental health first aid, participants with favourable baseline scores enroled in this study. Research that had a much longer time frame for recruiting participants and could engage interested parties in more subtle ways, may have resulted in a different sample composition.

A third limitation of this research is the size of the sample who reported providing a first aid intervention. Although the proportion of participants who reported providing some form of help (*n* = 31, 42%) was similar to previous evaluations of MHFA training [e.g. [56], the power to uncover a statistically significant effect is limited by such small numbers. Importantly, the only investigation to examine first aid experiences in a follow-up period longer than six months, found that 78% of respondents had experienced a situation in which they had provided some help, 19 to 21 months after receiving the training [55]. To ensure sufficient statistical power in future research evaluating MHFA training, a combination of a larger number of trained participants and a longer follow-up period should be employed.

### Implications of this research

Across most measures of knowledge, significant improvements were found from baseline to post-training. However, these changes were often not maintained at follow-up, as sharp declines occurred in the follow-up period. That the current research was not able to find sustained effects for a number of instruments suggests that the duration or ‘dosing’ effect of the training was inadequate. Given that evaluations of the full MHFA training program, which is presented across four sessions and totals 12 hours, has found much stronger effects at six month follow-up, it would be beneficial for the field of eating disorders for future research to investigate the effect of providing the current training within the full MHFA syllabus. It is also possible that presenting the current training over two or three sessions, with the inclusion of more material focused on the reduction of stigmatising attitudes and modeling of first aid behaviours, would produce lasting significant changes to knowledge, attitudes and behaviours. Such change would no doubt be important in encouraging those with eating disorders to seek appropriate help early, and thereby reduce the current heavy burden associated with these illnesses.

## Conclusions

Despite the short duration of the training’s impact, this research has provided preliminary evidence that mental health first aid training for eating disorders is associated with an increase in accurate recognition of eating disorders, knowledge of effective treatments and consensus-based first aid strategies, and confidence in providing help. The training was also associated with appropriate treatment seeking and appropriate first aid strategies, in a number of first aid interventions provided by participants to individuals with suspected eating disorders. Furthermore, there was no evidence that the training had a negative impact on the mental health of participants or the first aid they provided to others. Future research is needed to elucidate the optimal duration of the training program to achieve sustained increases in knowledge and attitudes, and to assess whether significant changes in first aid behaviour emerge over time. The current investigation, however, can confirm that the concept of mental health first aid can be usefully applied to improve mental health literacy and help-seeking behaviours for the eating disorders.

## Competing interests

AFJ is the scientific director of of Mental Health First Aid International.

## Authors’ contributions

This research was completed towards the fulfillment of the degree of doctor of philosophy for LMH. AFJ was the primary, and SJP was the secondary, academic supervisor. All authors made substantial contributions to the conception and design of the research, and to the analysis and interpretation of data. All authors were involved in drafting and revising this manuscript and have given this version final approval to be published.

## Pre-publication history

The pre-publication history for this paper can be accessed here:

http://www.biomedcentral.com/1471-244X/12/98/prepub

## Supplementary Material

Additional file 1 **Document 1.** Results from the First Aid Experiences Questionnaire. This file contains a description of the qualitative data collected using the First Aid Experiences Questionnaire, which assessed the nature of the first aid interventions provided by participants who attended the mental health first aid training for eating disorders program. Click here for file
