# Supplementary material for: Mental health first aid for eating disorders: pilot evaluation of a training program for the public
Source: BMC Psychiatry. 2012 Aug 2;12:98. doi: 10.1186/1471-244X-12-98 (PMC3549729; doi:10.1186/1471-244X-12-98)
Supplement: Additional file 1 — Document 1. Results from the First Aid Experiences Questionnaire. This file contains a description of the qualitative data collected using the First Aid Experiences Questionnaire, which assessed the nature of the first aid interventions provided by participants who attended the mental health first aid training for eating disorders program. [file 1471-244X-12-98-S1.doc]

# Additional Document 1

**Results from the First Aid Experiences Questionnaire**

To assess the qualitative nature of first aid interactions participants had with individuals with eating disorders, the *First Aid Experiences Questionnaire* asked a number of open-ended questions about their intervention. Of the 73 participants in the total sample, 41% (*n* = 30) of participants provided feedback on their first aid experiences. A series of questions about whether the participant had been able to assist the person and how they felt the intervention had gone, were then presented to these participants. Those who did not report on a first aid experience (*n* = 43, 59%) were asked a series of open-ended questions about what they would do in the future if a situation arose.

Participants who did not experience a first aid situation (n = 43)

When asked *Is this what you would have expected, or is it somewhat surprising not to have come across such a situation?*, 17 participants indicated that they were surprised, whereas another 17 participants responded that they had expected not to encounter someone with an eating disorder. Eight participants mentioned that they may have been in contact with someone with an eating disorder, but were not aware of the illness because their friendship was not intimate enough to detect symptoms (e.g. *I believe there is likely to be someone around me who would have a problem like this, but most likely not someone close enough for me to notice the subtle changes*). Furthermore, three participants mentioned that prior to the course they had contact with an individual with an eating disorder and that this contact has remained unchanged in the last six months.

Next, participants were asked *In the future, if you were to come across someone who you believed was experiencing an eating disorder, how well prepared would you feel to deal with the situation?.* In response, 33 participants indicated that they felt prepared, the majority indicating that they were ‘well’ or ‘very well’ prepared. One participant noted that they did not feel at all prepared. Nine participants mentioned that they felt more prepared as a result of the training. Five participants mentioned feeling as though they had all the information and knowledge required to assist, but remained uneasy about the prospect of approaching someone about their concerns (e.g. *I would feel quite prepared, but there is no rule that says how every individual is going to act when they have an eating disorder, so there will always be an element of feeling unprepared*).

Finally, participants were asked *How has attending 'Mental Health First Aid Training Course for Eating Disorders' changed how you relate to or feel about people who experience eating disorders?.* Fifteen reported feeling as though they knew more about eating disorders. Fourteen reported knowing more about how to assist someone, or feeling more confident in providing assistance. Interestingly, 14 participants reported feeling that as a result of the training they felt more empathy towards those with eating disorders and had a greater understanding of the distress experienced (e.g. *It has given me cause to have a little more sympathy and compassion for people with [these] disorders. I used to view it a little as a sign of weakness, but really it is more than that*). Finally, eight participants mentioned that it had not changed their knowledge or feelings towards people with eating disorders, two of whom qualified their response by indicating that they had a high level of knowledge and experience prior to the training.

Participants who experienced a first aid situation (n = 30)

Among those who had contact with someone with a suspected eating disorder, 70% (*n* = 21) were students and 30% (*n* = 8) were staff. Of the 69 students in the sample, 30% had contact with someone with a suspected eating disorder, and of the 21 staff in the sample, 38% had. A chi-square analysis found no significant difference between the proportion of students and staff who had encountered a first aid situation χ^2^(1) = .001, *p* = .98.

To assess the nature of the first aid experience, participants were first asked *Could you tell us something about the situation(s) and the problem(s) you believed the person was experiencing?* In response, 15 participants described that they had witnessed a change in eating habits, with a particular focus on intake restriction (e.g. *Only eating very small portions of very healthy foods, such as salad*). Binge eating was mentioned by two participants. Eleven participants mentioned witnessing a change in exercise, or a belief that exercise was excessive (e.g. *Has to exercise every day*). Vomiting was mentioned once. Seventeen participants mentioned a change in weight, however all comments were about weight loss and none mentioned witnessing fluctuations in weight or weight gain (e.g. *Noticed massive weight loss*).

Participants were then asked *Did you try to assist the person you thought might be developing or experiencing an eating disorder?.* Those who responded ‘yes’ (*n* = 20) were then given a series of questions about what assistance had been given. Those who responded ‘no’ (*n* = 10) were asked about the circumstances leading to assistance not being provided.

*No assistance given (n = 10)*

In response to the question *What was the reason(s) that you were not able to assist that person?*, five participants mentioned that they felt they were not close enough to the person to provide the first aid and that there were other individuals who were more appropriate, because of an existing relationship. Four participants mentioned that the person was already receiving adequate care from others and one mentioned that their offer of assistance had been refused.

*Assistance provided (n = 20)*

In response to the item *Can you give us an example of something you did to assist the person?,* it was expected that participants would list some or all of the actions in the ALGEE action plan (see Figure 1). Given that it is not necessary to complete the entire action plan, depending on the situation or the person’s problem, it was not expected that all participants would list all actions. However, the first two; *A – Approach the person, assess and assist* and *L - Listen non-judgmentally*; are primary actions, which should be completed each time mental health first aid is provided. Eleven participants mentioned approaching the person and having a discussion about their concerns. A further seven participants mentioned that they had discussed their concerns with an individual who would be a more appropriate person than themselves to provide first aid. This was most often a peer who was seen to have a closer friendship (e.g. *I brought the problem to the attention of her closer friends, and asked them to keep an eye on her [as] I'm not close enough for either of us to be comfortable with me talking to her*) or an authority figure within the residential college who had a duty of care (e.g. *Informed the head of pastoral care at my college of the situation*). Two participants mentioned that they had offered information and resources to the person about eating disorders, six mentioned encouraging the person to seek professional help and five mentioned encouraging other supports (e.g. *I tried spending more time with her, encouraged her to come to meals with me*)*.*

Participants were then asked to respond to the question *When assisting the person did you use the information provided in the 'Mental Health First Aid Training Course for Eating Disorders'?* using the forced-choice options. Fourteen responded ‘yes’, none ‘no’ and six ‘not sure’. The next item *How successful do you think you were in assisting the person?* used a 5-point Likert scale, listing options from ‘very successful’ to ‘very unsuccessful’. However, only two of the response options were used by participants; ‘successful’ (*n* = 9) and ‘neither successful nor unsuccessful’ (*n* = 11). This item was followed by an open response question *Would you like to comment on what happened?* to which 13 participants provided responses. Two mentioned they were unaware of the outcome because they were not the person providing the first aid. Three mentioned that the person had received professional help, three that the person had started to recover, and three that they felt the person had started to make some changes but were unsure how much recovery had progressed. In addition to questions about success, participants were asked *Do you think the information in the 'Mental Health First Aid Training Course for Eating Disorders' contributed to the level of success you had in assisting the person?.* In response, six participants said ‘very much’, 12 said ‘a little’, and one each said ‘not sure’ and ‘not really’.

When asked *When assisting the person, did you do anything differently from what you would have done before attending the 'Mental Health First Aid Training Course for Eating Disorders'?,* 11 participants responded ‘yes’, four responded ‘no’ and five responded ‘not sure’. The 11 who responded ‘yes’ provided details about how they had changed. Of these, seven mentioned that they changed the way they approached the person (e.g. *I changed how, when, and where I approached the individual*) or that before they wouldn’t have approached the person at all (e.g. *I approached them, which is something I might not have done without the knowledge of the first aid training*). Four participants noted that because they were more knowledgeable about eating disorders, they noticed more symptoms. Three noted that they felt comfortable suggesting appropriate professional help, and two said they were able to be more empathic and supportive of the person than before.

Participants were also asked if they had encouraged the person to seek professional help. A large majority (*n* = 10) said that they had suggested it, three said they had not, four said they were ‘not sure’ and three mentioned that the person they were assisting was already seeking appropriate help. When asked if the person had sought help as a result of their suggestion, seven participants responded ‘yes’, seven said ‘not sure’, and six ‘not applicable’.

Assistance with other mental health problems

To assess whether the information particular to eating disorders provided in the training generalised to providing assistance to individuals with other mental health problems, the First Aid Experiences Questionnaire also asked *Since completing the ‘Mental Health First Aid Training Course for Eating Disorders’ have you tried to assist someone you thought might be developing or experiencing a mental illness or a mental health crisis, other than an eating disorder?*. Twenty-one participants (29%) said ‘yes’. Among those, 62% (*n* = 13) were students and 38% (*n* = 8) were staff. Of the 69 students in the sample, 19% had contact with someone with a suspected mental health problem, and of the 21 staff in the sample, 38% had. A chi-square analysis found no significant difference between the proportion of students and staff who had encountered a possible first aid situation χ^2^(1) = 1.7, *p* = .19. Thirteen (62%) of the participants who assisted someone with a suspected mental health problem had also assisted someone with a suspected eating disorder.

When asked what problem or crisis participants thought the person they were assisting might have been experiencing, 10 said depression, four said anxiety and depression, four mentioned recovering from grief or a traumatic experience and two mentioned suicide or self-harm. Schizophrenia and substance abuse were each mentioned once.

Participants were also asked to provide examples of how they were able to assist. Fifteen mentioned talking to the person, assessing their wellbeing and level of risk, or assisting them to recover or manage (e.g. *Listened for a while, made certain that she no longer felt any suicidal thoughts, and offered any help I could give regarding the problems she was experiencing*)*.* Nine participants mentioned trying to reassure the person and provide emotional support (e.g. *I tried to take the stigma off the problem, and 'normalise' it in a sense but still emphasise that it's serious and it's good to seek help - she was in the process of seeking help for it so I just reaffirmed this*). Suggesting that the person seek professional help was also common among participants’ strategies for providing assistance, as nine also mentioned this. In addition, eight participants mentioned trying to encourage the person to seek out other supports (e.g. *Asked them what things I could do around the house that would support them feeling better. We also began to make plans to do social outings to look forward to*).

In response to the question *When assisting the person did you use the information provided in the 'Mental Health First Aid Training Course for Eating Disorders'?* seven participants responded ‘yes’, nine responded ‘no’ and five ‘not sure’. In response to the question *How successful do you think you were in assisting the person?*, three participants responded ‘very successful’, 14 responded ‘successful’, three responded ‘neither’ and one ‘not successful’. As was the case with assisting an individual with an eating disorder, when asked to comment on how they felt about the success of their assistance, participants frequently mentioned the ongoing nature of the person’s recovery (*n* = 7). Four participants also mentioned how they believed the person had felt supported by their intervention (e.g. *She really trusted me and my opinions and I think she felt less alone knowing I was there for her*) and an additional four mentioned that the person was seeking ongoing professional help.

In response to the question *When assisting the person, did you do anything differently from what you would have done before attending the 'Mental Health First Aid Training Course for Eating Disorders'?,* four participants responded ‘yes’, 10 responded ‘no’ and seven responded ‘not sure’. Those who responded ‘yes’ provided details about how they had changed. Of these, three mentioned that they assessed for level of suicide risk where they would not have done so before (e.g. *I flat-out asked this person if they had considered suicide. I did not simply take control of the situation, but tried to indicate to the person that their issues were serious and concerned me greatly*). In addition, three participants noted that they felt the training had helped them to regulate their own response to the person’s situation in a more helpful and supportive way (e.g. *I assessed how serious the situation was and made sure not to freak out or become overly aware and paranoid about possibly symptomatic behaviours*). One participant mentioned that although they had not done anything differently to before, the training reassured them that they had previously been doing the ‘right’ things.

When asked if they had encouraged the person to seek professional help, 11 said ‘yes’, one said ‘no’ and nine responded that the person they were assisting was already seeking appropriate help. When asked if the person had sought help as a result of their suggestion, nine participants responded ‘yes’, four said ‘no’, one ‘not sure’, and seven ‘not applicable’.
